# Fazekas scale magnetic resonance imaging assessment in Alzheimer’s disease and primary age-related tauopathy

**DOI:** 10.1007/s00234-024-03464-2

**Published:** 2024-09-26

**Authors:** Miguel Quintas-Neves, Francisco C. Almeida, Kathryn Gauthreaux, Merilee A. Teylan, Charles N. Mock, Walter A. Kukull, John F. Crary, Tiago Gil Oliveira

**Affiliations:** 1 School of Medicine, Life and Health Sciences Research Institute (ICVS), University of Minho, Braga, Portugal; 2 ICVS/3B’s—PT Government Associate Laboratory, Braga/Guimarães, Portugal; 3 Department of Neuroradiology, Unidade Local de Saúde de Braga, Braga, Portugal; 4 Department of Neuroradiology, Centro Hospitalar Universitário Do Porto, Porto, Portugal; 5 Department of Epidemiology, National Alzheimer’s Coordinating Center, University of Washington, Seattle, WA U.S.A.; 6 Neuropathology Brain Bank & Research Core, Department of Pathology, Nash Family Department of Neuroscience, Department of Artificial Intelligence, & Human Health, Friedman Brain Institute, Ronald M. Loeb Center for Alzheimer’s Disease, Icahn School of Medicine at Mount Sinai, New York, NY U.S.A.

**Keywords:** PART = Primary age-related tauopathy, AD = Alzheimer’s disease, CERAD = Consortium to Establish a Registry for Alzheimer’s disease

## Abstract

**Background:**

Brain vascular pathology is an important comorbidity in Alzheimer’s disease (AD), with white matter damage independently predicting cognitive impairment. However, it is still unknown how vascular pathology differentially impacts primary age-related tauopathy (PART) compared to AD. Therefore, our objectives were to compare the brain microangiopathic burden in patients with PART and AD, evaluated by MRI, while assessing its relation with neuropathological findings, patterns of brain atrophy and degree of clinical impairment.

**Methods:**

Clinical information, brain MRI (T1 and T2-FLAIR) and neuropathological data were obtained from the National Alzheimer’s Coordinating Centre ongoing study, with a total sample of 167 patients identified, that were divided according to the presence of neuritic plaques in Consortium to Establish a Registry for Alzheimer’s disease (CERAD) 0 to 3. Microangiopathic burden and brain atrophy were evaluated by two certified neuroradiologists, using, respectively, the Fazekas score and previously validated visual rating scales to assess brain regional atrophy.

**Results:**

Significant correlations were found between the Fazekas score and atrophy in the fronto-insular and medial temporal regions on both groups, with PART showing overall stronger positive correlations than in AD, especially in the fronto-insular region. For this specific cohort, no significant correlations were found between the Fazekas score and the degree of clinical impairment.

**Conclusion:**

Our results show that PART presents different pathological consequences at the brain microvascular level compared with AD and further supports PART as an independent pathological entity from AD.

**Supplementary Information:**

The online version contains supplementary material available at 10.1007/s00234-024-03464-2.

## Introduction

Primary age-related tauopathy (PART) is a neuropathological condition, characterized by the postmortem finding of tau-positive neurofibrillary tangles in the brain, without neuritic plaque deposition (“definite” PART). Neurofibrillary tangle neuropathological classification is based on Braak staging, while amyloid-beta neuritic plaque pathology is classified by the Consortium to Establish a Registry for Alzheimer’s disease (AD) (CERAD) staging, which in “definite” PART is 0, corresponding to the absence of neuritic plaques [1]. On the other hand, AD is diagnosed by the identification of both neurofibrillary tangles and amyloid-beta brain deposition, whether in vivo [using the so-called AT(N) biomarkers proposed by the National Institute on Aging and Alzheimer’s Association (NIA-AA) 2018 Research Framework [2]] or postmortem [using the “ABC score”, by grading amyloid plaques with Thal phases (A), neurofibrillary tangles with Braak staging (B) and neuritic plaques with CERAD assessment (C)] [3].

Several risk factors have been described for AD, such as genetic, age, traumatic brain injury or vascular disease [4]. Interestingly, cerebrovascular disease burden in the form of white matter T2 hyperintensities in brain MRI has been found to be a predictor of AD progression and can be clinically assessed by the Fazekas grading score [5]. This is a three-point based scale [6] that independently rates deep and periventricular white matter lesions related to leukoaraiosis, with the former being frequently associated with small vessel disease. Moreover, it is known that the amount of amyloid-beta deposition and white matter lesions independently predict cognitive impairment, which supports the diagnostic usefulness of assessing white matter damage [7]. Recently, a neuropathological study found a significant correlation between cognitive impairment and cerebrovascular disease in PART patients [8]. Despite the known importance of brain vascular pathology as a comorbidity in AD, it is still underexplored how brain vascular lesion burden differentially affects PART compared to AD, in terms of cognition and brain atrophy, namely by using clinically applicable visual rating scales [9–11]. Therefore, our study aimed to assess how PART and AD are differentially impacted by brain microangiopathy burden, evaluated by in vivo MRI.

This article follows the STROBE reporting guidelines.

## Materials and methods

### Study design, participants and selection criteria

This is a retrospective study based on data obtained from the National Alzheimer’s Coordinating Center, a repository for data collected at the Alzheimer’s Disease Centers located across the United States of America. Each Alzheimer’s Disease Centers collected standardized clinical data via the Uniform Data Set and neuropathological evaluations obtained at autopsy to the Neuropathology Data Set. Both datasets have been described in detail elsewhere [12–16]. Our sample was obtained from the September 2019 data freeze (n = 38,836 patients), that included 4192 patients clinical Uniform Data Set data within 2 years from death and neuritic plaque burden assessed at autopsy. All Uniform Data Set visits from patients with MRI scans performed no more than 4 years before the date of death and who had neuropathology data available were collected (n = 334 patients); only the last Uniform Data Set visit and respective brain MRI scan were considered for the analysis. Participants with the following comorbidities were excluded: (a) neuropathological evidence of frontotemporal lobar degeneration, amyotrophic lateral sclerosis, prion disease, or argyrophilic grains; (b) with clinical evidence of dementia with Lewy bodies, Parkinson disease, Down syndrome, Huntington disease, prion disease, corticobasal degeneration, or progressive supranuclear palsy; (c) with other brain lesions that biased atrophy assessment (e.g., brain tumor, brain herniation, vascular malformation, lymphocytic meningoencephalitis, traumatic brain injury, demyelinating disease, large territorial ischemic lesion). After applying these exclusion criteria, participants with no T2-FLAIR sequence available on brain MRI were also excluded, after which a final sample of 167 participants was obtained. PART cases were defined as having no neuritic plaques (CERAD 0), that is, “definite” PART.

### Neuropathology data

This information was collected by the Alzheimer’s Disease Centers by the use of a standardized Neuropathology Form on those patients who died and consented to autopsy and neuropathologic examination. Afterwards, participants were categorized according to the Braak stage for neurofibrillary degeneration (i.e. neurofibrillary tangles distribution) and CERAD stage (i.e. neuritic plaques density). Details on brain tissue preparation and staining within the National Alzheimer’s Coordinating Center Neuropathology dataset have been previously described [13].

### Brain MRI data

MRI examinations were performed on 1.5 T or 3 T scanners, both from Philips, Siemens or GE manufacturers. Despite different protocols between centers, for our imaging analysis we only used 3D T1-weighted acquisitions (in order to grade the degree of regional brain atrophy) and 2D T2-FLAIR sequences (in order to grade leukoaraiosis).

### Imaging analysis

In order to assess brain atrophy, we used a previously validated visual rating scale [9] that takes into account the following 6 regions: anterior cingulate, orbito-frontal, anterior temporal, fronto-insular, medial temporal, and posterior. We defined atrophy as a cross-sectional concept, corresponding to the score attributed on the basis of the aforementioned rating scales. As already described by the simplified version: orbito-frontal and anterior cingulate regions were both rated on the first anterior coronal slice where the corpus callosum becomes visible; the fronto-insular was rated over three slices, starting on the first anterior coronal slice where the anterior cingulate becomes visible and moving posteriorly; the anterior temporal was rated at the level of the temporal pole, immediately anterior to where the “temporal stem” connects the frontal and temporal lobes; the medial temporal was rated according to the medial temporal lobe atrophy score [17], performed on the hippocampus at the same coronal section of the anterior pons; the posterior region was rated according to a four-point posterior atrophy scale described by Koedam [18], being the overall score based on the presence of atrophy in sagittal (widening of the posterior cingulate and parieto-occipital sulcus, and atrophy of the precuneus on both sides by considering paramedian sagittal sections), as well as axial and coronal (widening of the posterior cingulate sulcus and sulcal dilatation in parietal lobes) sections, assessed for left and right separately [9]. For each brain region scale, an average of both hemispheres was calculated. In order to aid and increase acuity of the rating process, reference imagens for each rating scale were provided to the classifiers based on Harper et al. [9].

In order to assess leukoaraiosis, the Fazekas scale was used [6], considering separately the periventricular and the deep white matter, according to a rating system ranging from 0 (none) to 3 (severe), as originally described. In order to aid and increase the acuity of the rating process, reference images for the deep and periventricular Fazekas scales were provided to the classifiers [19].

**Fig. 1:**
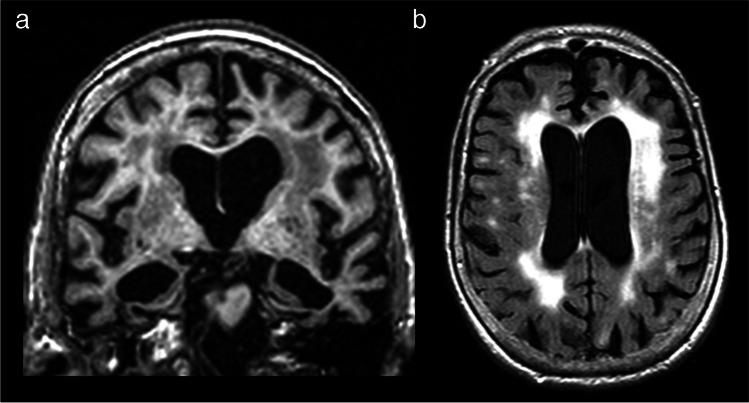
Representative case of a 91 year-old male patient with AD. (**a**) Coronal reformat of a 3D T1-weighted sequence shows severe widening of the choroid fissures and severe enlargement of the temporal horns, with marked atrophy of both hippocampi (medial temporal lobe atrophy – MTA – score of 4). (**b**) Axial T2-FLAIR sequence reveals hyperintensities involving the periventricular and the deep white matter, with irregular periventricular signal extending to the deep white matter in the former and severe confluence in the latter (periventricular and deep Fazekas score of 3)

Two independent classifiers (unaware of the clinical diagnosis) with 11 and 7 years, respectively, of experience in clinical neuroradiology were responsible for rating the images. In all cases, an average of both classifiers was used. Figure 1 depicts an example of a patient in which this rating method was performed.

### Neuropsychological assessment

Local Alzheimer’s Disease Centers assessed participants using the CDR and the Uniform Data Set version 2 neuropsychological test battery [15]. We used the CDR Sum of Boxes (CDR-SB) performed at the last Uniform Data Set visit prior to death. The Washington University CDR was reviewed by Morris and collaborators in 1993 [20] with the purpose of staging the severity of AD, and takes into consideration six cognitive categories: orientation, memory, judgment and problem solving, community affairs, home and hobbies, and personal care; it is based on a five-point scale in which none = 0, questionable = 0.5, mild = 1, moderate = 2, and severe = 3. The CDR-SB is calculated by summing the ratings of the previously mentioned six cognitive domains and, therefore, reflects a quantitative global measure, that ranges from 0 (normal) to 18 (severe dementia) [21].

### Statistical analysis and *bias* control

Part of our analysis was based on the division into four groups according to the CERAD score (ie, density of neocortical neuritic plaques): CERAD 0 (none – “definite” PART); CERAD 1 (sparse – mild AD); CERAD 2 (moderate – moderate AD); CERAD 3 (severe – severe AD). One-way ANOVA was performed in order to assess potential differences between groups in terms of baseline characteristics, followed by Tukey post hoc analysis or χ^2^ test whenever these characteristics were significantly different (*p* < 0.05). Intraclass correlation coefficients were calculated for checking the acuity of the rating process between both classifiers per each region evaluated for atrophy and for the Fazekas grading (periventricular and deep), with significant correlations obtained between classifiers for each variable (Online Resource 1), and overall good reliability (> 0.75). One-way ANOVA was also performed in order to check for statistically significant differences between Fazekas grading in the 4 groups of CERAD, followed by Tukey post hoc analysis whenever significant results were obtained (*p* < 0.05), without correcting for other factors and after correcting for Braak and age; the same method was applied to check differences between groups with absence (i.e. “definite” PART) and presence (i.e. AD neuropathological change) of neuritic plaques.

Linear regression models and Pearson correlation coefficients were used in order to assess the relation between the Fazekas score (periventricular and deep) and the CDR-SB, uncorrected and after correction for age and Braak, in “definite” PART versus AD; values were expressed as (*R*) and considered statistically significant when *p *< 0.05. Linear regression models and Pearson correlation coefficients were also used to assess the relation between the Fazekas score (periventricular and deep) and the percentage of relative atrophy in the several evaluated brain regions, after correction for age. ANCOVA was then performed in order to compare the slopes of the regression lines obtained. All statistical analyses and graphical representation were performed on SPSS Statistics version 29 and GraphPad software version 10.0.0.

## Results

167 participants were included: 29 with no (“definite” PART – CERAD 0), 17 with sparse (CERAD 1), 54 with moderate (CERAD 2), and 67 with severe (CERAD 3) neuritic plaques density. CERAD 0, 2 and 3 showed a male predominance (more than 50%), while CERAD 1 had a female predominance (approximately 65%); there was, however, no significant differences in gender between groups (Table 1). Mean age at death and mean age at last MRI were significantly different between groups, with lower values on CERAD 0 (78.1 ± 12.4 and 76.0 ± 12.9 years old, respectively) and 3 (80.4 ± 8.5 and 77.8 ± 8.7 years old, respectively) and higher values on CERAD 1 (88.8 ± 6.1 and 86.8 ± 5.4 years old, respectively) (Table 1). The global CDR and CDR-SB were significantly lower for CERAD 1 (0.8 ± 0.9 and 4.5 ± 5.2, respectively) and 2 (1.0 ± 0.7 and 5.2 ± 4.2, respectively), and higher for CERAD 3 (1.4 ± 0.7 and 8.4 ± 4.5, respectively); moreover, CERAD 0 presented values of CDR between those of CERAD 2 and 3, with no statistically significant differences between them. Braak staging was also significantly different between groups, with a predominance of lower grades (Braak I and II) in PART and higher grades (Braak V and VI) in the more advanced AD spectrum CERAD 3 cases (Table 1).

**Table 1:** Characterization of patients according to the density of neocortical neuritic plaques (CERAD score)

	PART	AD			
	CERAD 0 (None) (n = 29)	CERAD 1 (Sparse) (n = 17)	CERAD 2 (Moderate) (n = 54)	CERAD 3 (Severe) (n = 67)	***p***
Male sex, n (%)^a^	19 (65.5)	6 (35.3)	30 (55.6)	45 (67.2)	0.087
Age at death, mean (SD)^b^	78.1 (± 12.4)	88.8 (± 6.1)	83.1 (± 8.4)	80.4 (± 8.5)	< 0.001
Age at last MRI, mean (SD)^c^	76.0 (± 12.9)	86.8 (± 5.4)	81.0 (± 8.5)	77.8 (± 8.7)	< 0.001
Age MRI-Death, mean (SD)^d^	2.1 (± 1.1)	2.0 (± 1.4)	2.1 (± 1.2)	2.5 (± 1.1)	0.118
CDR-SB^e^	6.1 (± 6.3)	4.5 (± 5.2)	5.2 (± 4.2)	8.4 (± 4.5)	< 0.001
Global CDR^e^	1.2 (± 1.1)	0.8 (± 0.9)	1.0 (± 0.7)	1.4 (± 0.7)	0.003
Braak stage, n (%)^f^					0.000
None	6 (20.7)	0 (0.0)	1 (1.9)	0 (0.0)	
I	10 (34.5)	1 (5.9)	1 (1.9)	0 (0.0)	
II	8 (27.6)	6 (35.3)	9 (16.7)	0 (0.0)	
III	3 (10.3)	7 (41.2)	12 (22.2)	2 (3.0)	
IV	2 (6.9)	1 (5.9)	11 (20.4)	7 (10.4)	
V	0 (0.0)	2 (11.8)	12 (22.2)	21 (31.3)	
VI	0 (0.0)	0 (0.0)	8 (14.8)	37 (55.2)	
Braak stage, mean (SD)	1.5 (± 1.2)	2.8 (± 1.1)	3.8 (± 1.5)	5.4 (± 0.8)	< 0.001

^a^Male sex refers to the absolute mean and relative percentage of male patients in a given group, represented as n (%)

^b^Age at death is the subject age at the time of death

^c^Age at last MRI is the subject age at the time the last MRI was performed

^d^Age MRI-Death is the difference between the subject age at the last performed MRI and time of death. These three variables are reported in years as a continuous variable with mean and standard deviation (SD)

^e^CDR-SB refers to the sum of boxes score from the CDR® Dementia Staging Instrument and global CDR refers to the global Clinical Dementia Rating score; they are both attributed to the subject in the last clinical visit and are also reported as continuous variables with mean and standard deviation

^f^For each Braak stage (from none to VI) values are represented as number of cases and percentage of total. Data presented as n (%) and mean (± SD)

*p* value for One-way ANOVA or chi-square test, as appropriate. *p* < 0.05 considered as significant

**Fig. 2:**
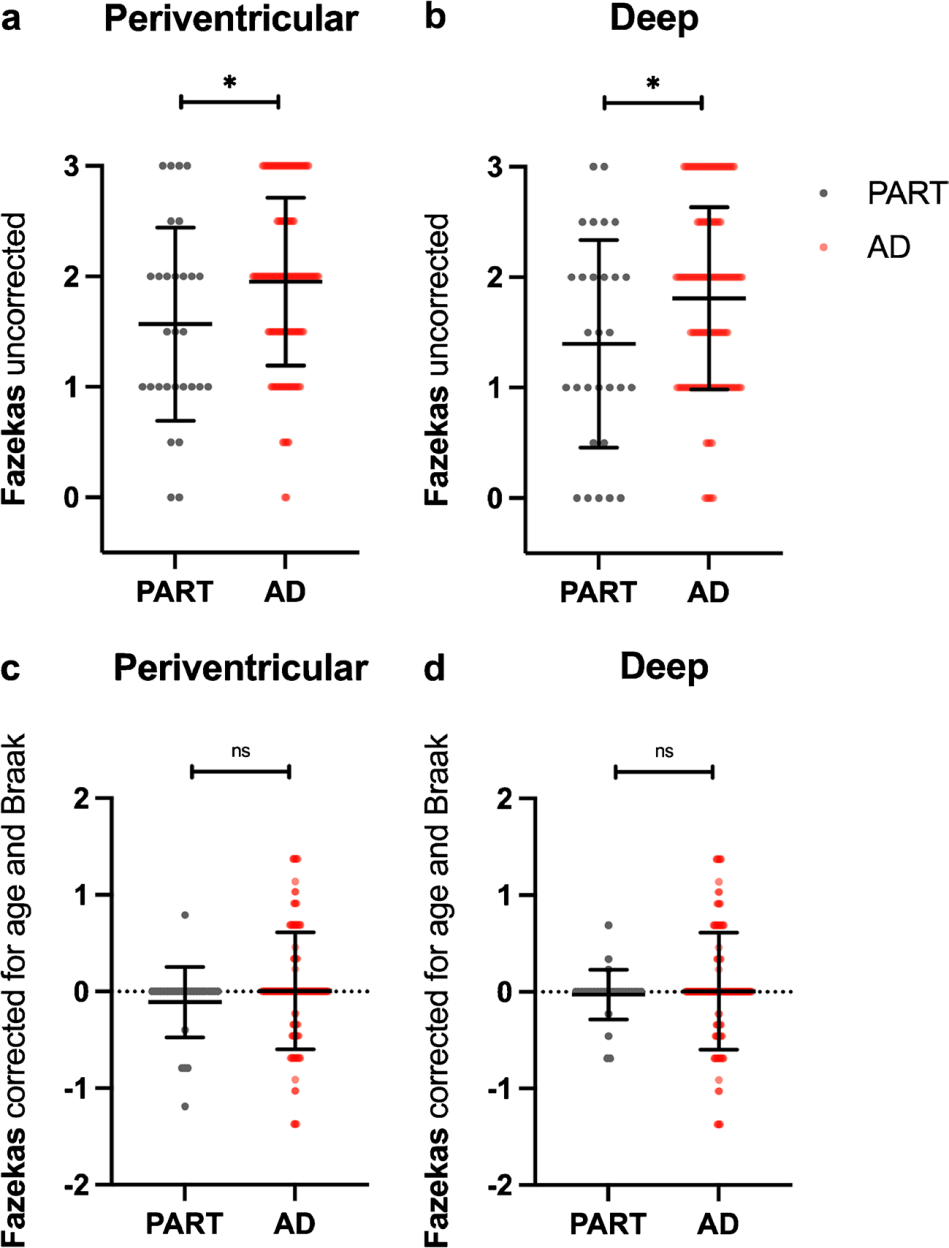
No significant differences were found in the Fazekas grading between patients with PART and AD. Uncorrected (**a**, **b**) and standardized residuals for age and Braak (**c**, **d**) for deep and periventricular Fazekas scores among patients with absence (i.e. PART) or presence (i.e. AD) of neocortical neuritic plaques after neuropathological evaluation. * *p *< 0.05. ‘ns’ represents non-significant differences between groups

No statistically significant differences were found in the periventricular or deep Fazekas scores considering groups from CERAD 0 to 3, without correction for potential confounding factors (*p* = 0.116 and 0.132, respectively) and after correction for age and Braak (*p* = 0.059 and 0.311, respectively) (Online Resource 2). Although statistically significant differences were found in periventricular and deep Fazekas scores between “definite” PART (CERAD 0) and the AD spectrum (CERAD 1–3) (*p* < 0.05 – Fig. 2), no significant differences remained after correcting for age and Braak (*p* = 0.182 and 0.634, respectively).

No significant correlations were found between the Fazekas scores (periventricular or deep) and the CDR-SB on both “definite” PART and AD participants, without correction for other factors and after correcting for age and Braak (Online Resource 2).

**Fig. 3:**
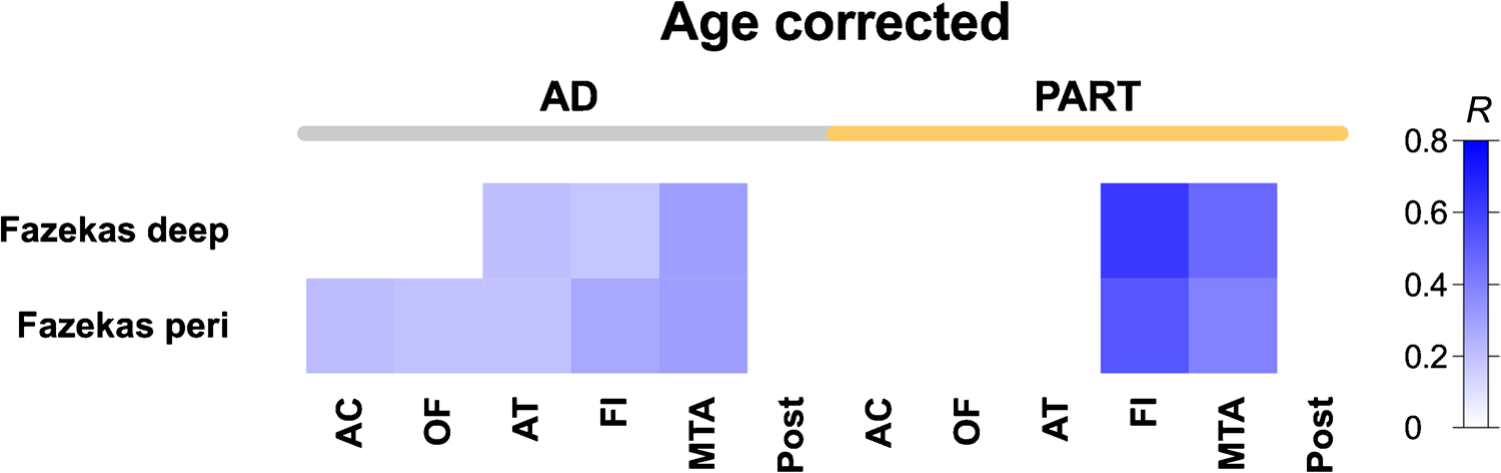
Correlation analysis between relative regional brain atrophy and Fazekas scores, corrected for age, shows differential patterns in AD and PART. Pearson correlation analysis of relative brain atrophy residuals after linear regression with age per region versus Fazekas scores (periventricular and deep) among 2 groups of participants, distributed according to the absence (i.e. PART) or presence (i.e. AD) of neocortical neuritic plaques after neuropathological evaluation. The regions evaluated are anterior cingulate (AC), orbito-frontal (OF), fronto-insular (FI), medial temporal (MTA) and posterior (Post). Only showing pairs with *p* < 0.05 in the correlational analysis. Color indicates *R* Pearson coefficient

In AD patients, significant positive correlations (*p* < 0.05) were found between the Fazekas score (periventricular and deep) and atrophy in the medial temporal (*R* = 0.30 for both), anterior temporal (*R* = 0.19 and 0.20, respectively) and fronto-insular regions (*R* = 0.27 and 0.18, respectively), and between the periventricular Fazekas and the orbito-frontal (*R* = 0.19) and the anterior cingulate (*R* = 0.21) regions, while in PART patients, significant positive correlations (*p* < 0.05) were found between the Fazekas score (periventricular and deep) and atrophy in the medial temporal (*R* = 0.39 and 0.47, respectively) and fronto-insular regions (*R* = 0.53 and 0.62, respectively) (Fig. 3). Moreover, a significant difference was found (*p* < 0.05) between the regression lines assessing atrophy in the fronto-insular region and the deep Fazekas score in AD versus PART patients (Online Resource 1).

## Discussion

Previous evidence suggested that AD and PART could have differential cerebrovascular disease associated co-pathologies [8]. Using the Fazekas score as an MRI-based surrogate for leukoaraiosis, with the deep white matter hyperintensities predominantly reflecting small vessel disease, we observed no major statistically significant differences between groups of CERAD 0 to 3 or between “definite” PART and AD patients. However, significant correlations were found between the Fazekas score and regional atrophy patterns on both groups, with PART showing overall stronger positive correlations than in AD, particularly in the fronto-insular region. Even though cerebrovascular co-pathology, in this case represented by small vessel disease, does not substantially differ between PART and AD at the MRI level, it might be differentially contributing to regional brain atrophy. These results provide additional evidence that PART and AD might be two different entities, with potentially different pathological consequences at the brain microvascular level.

It was shown that significant cerebrovascular disease (co-defined as a Fazekas score ≥ 2) was a potential neurodegeneration driver in patients with no significant altered amyloid-beta pathology in PET (i.e. in suspected non-Alzheimer pathology – “SNAP”) [22]. Moreover, levels of amyloid deposition, as well as ratings of periventricular and deep Fazekas scores, have been found to discriminate between cognitively normal and demented individuals [7]. One of the described possibilities to potentially explain the link between cognitive impairment and periventricular white matter hyperintensities is the disruption of fibers in cognitive circuits across the brain, such as cholinergic projections from the basal forebrain to the cortex [23]. Moreover, elevated levels of activated microglia in periventricular white matter hyperintensities suggest an intrinsic neuroinflammatory response following the disruption of the blood–brain barrier [23]. This is not the case, however, for the subcortical/deep location [24], where white matter hyperintensities volume has been associated with lipid peroxidation in the blood, in the setting of hypertension, supporting the hypothesis of a vascular etiology in this location [25]. Adding to this, two recent studies [26, 27] found evidence that, in AD patients, white matter hyperintensities can develop in the setting of vascular disease and also secondary to AD pathology (i.e. AD-related white matter hyperintensities), with the latter potentially being explained by the following pathophysiological mechanisms: (a) axonal/Wallerian degeneration secondary to neurofibrillary tangles; (b) toxicity of amyloid-beta oligomers that can also lead to axonal degeneration; (c) neuroinflammation and microglial activation that could be involved in the pathogenesis and progression of AD. As such, AD-related processes, such as parenchymal/vessel amyloidosis and neurodegeneration, might be responsible for a large proportion of the increased white matter lesion volume found in AD and, therefore, the presence of elevated white matter hyperintensities in these patients may not necessarily reflect the presence of mixed vascular and AD pathologies [27], something future studies should better assess, possibly by correlating imaging findings (i.e. the Fazekas scores) with neuropathological findings at autopsy on those regions. It is also noteworthy that some literature favors a posterior predominance of white matter hyperintensities in AD (i.e. parieto-occipital and posterior periventricular areas) [28, 29], with the splenium of the corpus callosum being described as a potentially core feature of AD associated with worse cognition [26]. Future studies should evaluate this anteroposterior gradient and compare it to the commonly used Fazekas score (deep and periventricular), while also correlating with the number and type of basal ganglia lesions, as defined by the European Task Force on Age-Related White Matter Changes [30].

Even though we found no significant correlations between the Fazekas scores and the CDR-SB, which is arguably explained by the sample size, we observed significant correlations between the Fazekas scores and the degree of atrophy in several brain regions, further supporting the concept that cerebrovascular co-pathology, represented by leukoaraiosis and, most specifically, small vessel disease, might play an important role in the pathophysiology of PART and AD. Interestingly, the magnitude of correlation between microangiopathy and atrophy in the fronto-insular region was more pronounced in PART than in AD, which suggests that, in PART, atrophy patterns might be less relying on neuropathological changes than in AD. This is in line with previous evidence that showed a strong association between cerebrovascular disease and cognitive impairment in PART patients [8]. As PART cases with mild cognitive impairment or dementia are diagnosed as AD more than 50% of the time [31], these findings might contribute to a potential future distinction between these two entities at the clinical presentation level. We believe that by using validated visual rating scales that can be easily applied in a routine clinical setting might contribute to the progressive translation from the neuropathological diagnosis of PART to its clinical characterization.

This study has important limitations. It was based on a convenience, autopsy-based, sample, which limits its extrapolation. The National Alzheimer’s Coordinating Center database has inherent limitations of generalizability, given the participants tended to be more often caucasian and more affluent than the general population [32]. Moreover, in our sample, there was a tendency towards higher neuropathological burden in younger ages. Secondly, the use of visual rating scales, despite performed by trained neuroradiologists, is invariably associated with interobserver variability; however, in our case, significant correlations between both raters, with overall good interobserver agreement, was found. Also, the variability in MRI scanners and field strengths were potential sources of bias and the patients included in our sample had incomplete clinical information on other comorbidities that could be associated with brain atrophy and microangiopathy, such as hypertension or dyslipidemia, or on other neuropathologic features, such as TAR DNA-binding protein 43 pathology. Also, we did not assess other manifestations of small vessel disease, such as cerebral microbleeds, intracerebral hemorrhage, cortical superficial siderosis or lacunar infarcts, something future studies should tackle. Another limitation is that Braak staging is an incomplete measure of tau burden and, therefore, it would be beneficial to have other quantitative measures, such as tau PET. Finally, the retrospective nature of the study is a potential source of bias. Despite these limitations, the study has also major strengths, such as the use of multicentric data on a large group of individuals across the US, the persistently performed standardized process of collecting several clinical variables in every Uniform Data Set visit, the use of standardized neuropathological criteria to assess pathology at autopsy [33], and the unique nature of a database with antemortem clinically validated MRI sequences (T1 and T2-FLAIR) with gold-standard neuropathology postmortem diagnosis of PART and AD.

## Conclusions

Our study further supports the concept that PART might be a different neuropathological entity from AD, by showing different correlations between brain microangiopathy and atrophy in several brain regions, particularly in PART.

## Supplementary information

Below is the link to the electronic supplementary material.

Supplementary file1 (DOCX 25 KB)

## Data Availability

The data used in this study is available upon request on the National Alzheimer’s Coordinating Center database.
