# Supplementary material for: Fazekas scale magnetic resonance imaging assessment in Alzheimer’s disease and primary age-related tauopathy
Source: Neuroradiology. 2024 Sep 26;66(12):2185–93. doi: 10.1007/s00234-024-03464-2 (PMC11611984; doi:10.1007/s00234-024-03464-2)
Supplement: Supplementary file 1 — Supplementary file1 (DOCX 25 KB) [file 234_2024_3464_MOESM1_ESM.docx]

Supplementary tables

# **Table S1.** Correlation analysis between CDR Sum Of Boxes and the Fazekas scores in patients with Alzheimer’s disease and primary age-related tauopathy, before and after adjusting for age and Braak.

| **Uncorrected** | **AD** | | | | **PART** | | | |
| --- | --- | --- | --- | --- | --- | --- | --- | --- |
|  | **Fazekas perivent** | | **Fazekas deep** | | **Fazekas perivent** | | **Fazekas deep** | |
|  | *R* | *p* | *R* | *p* | *R* | *p* | *R* | *p* |
| CDR-SB | -0.08 | 0.384 | -0.11 | 0.184 | -0.15 | 0.443 | -0.16 | 0.404 |

| **Corrected age & Braak** | **AD** | | | | **PART** | | | |
| --- | --- | --- | --- | --- | --- | --- | --- | --- |
|  | **Fazekas perivent** | | **Fazekas deep** | | **Fazekas perivent** | | **Fazekas deep** | |
|  | *R* | *p* | *R* | *p* | *R* | *p* | *R* | *p* |
| CDR-SB | 0.01 | 0.948 | -0.13 | 0.125 | -0.03 | 0.893 | 0.10 | 0.606 |

The table shows the *R* Pearson correlation coefficients and respective *P*-values of the correlation analysis between the CDR Sum of Boxes (CDR-SB) and the Fazekas scores (periventricular and deep) among 2 groups of patients, distributed according to the absence (ie PART) or presence (ie AD) of neocortical neuritic plaques after neuropsychological evaluation. There are no significant correlations, nor differences between groups. *p* < 0.05 considered as statistically significant.

# **Table S2.** Correlation analysis between relative regional brain atrophy and the Fazekas scores corrected for age in patients with Alzheimer’s disease and primary age-related tauopathy.

| **Corrected age** | **AD** | | | | **PART** | | | |
| --- | --- | --- | --- | --- | --- | --- | --- | --- |
|  | **Fazekas perivent** | | **Fazekas deep** | | **Fazekas perivent** | | **Fazekas deep** | |
|  | *R* | *p* | *R* | *p* | *R* | *p* | *R* | *p* |
| AC | **0.21** | **0.016** | 0.13 | 0.140 | 0.20 | 0.293 | 0.28 | 0.148 |
| OF | **0.19** | **0.024** | 0.15 | 0.078 | 0.22 | 0.248 | 0.37 | 0.051 |
| AT | **0.19** | **0.026** | **0.20** | **0.021** | 0.07 | 0.724 | 0.22 | 0.246 |
| FI | **0.27** | **0.002** | **0.18*** | **0.031** | **0.53** | **0.003** | **0.62*** | **<0.001** |
| MTA | **0.30** | **<0.001** | **0.30** | **<0.001** | **0.39** | **0.037** | **0.47** | **0.010** |
| Post | -0.16 | 0.07 | -0.18 | 0.053 | -0.21 | 0.973 | 0.04 | 0.840 |

The analyses presented here are relating to figure 2. The table shows the *R* Pearson correlation coefficients and respective *P*-values of the correlation analysis between relative regional brain atrophy and the Fazekas scores (periventricular and deep) among 2 groups of patients, distributed according to the absence (ie PART) or presence (ie AD) of neocortical neuritic plaques after neuropsychological evaluation. As described in the manuscript, in AD patients, there are significant correlations (represented in **bold**) between the Fazekas scores (periventricular and deep) and atrophy in the medial temporal and orbitofrontal regions, and between the periventricular Fazekas and the fronto- insular region, while in PART patients, significant positive correlations were found between the Fazekas scores (periventricular and deep) and atrophy in the medial temporal and fronto-insular regions, and between the deep Fazekas and atrophy in the orbitofrontal region. Moreover, a statistically significant difference between the regression lines assessing atrophy in the frontoinsular region and the deep Fazekas score in AD versus PART patients (represented by *****). *p* < 0.05 considered as statistically significant.

# **Table S3.** Intraclass correlation coefficients (ICC) between raters.

|  | **ICC** | ***p*** |
| --- | --- | --- |
| Anterior Cingulate | 0.808 | <0.001 |
| Orbito-Frontal | 0.862 |  |
| Anterior Temporal | 0.687 |  |
| Fronto-Insular | 0.768 |  |
| Medial Temporal | 0.937 |  |
| Posterior | 0.817 |  |
| Periventricular Fazekas | 0.894 |  |
| Deep Fazekas | 0.910 |  |

The table shows significant intraclass correlations between both raters, with values between 0.5 and 0.75, 0.75 and 0.90, and greater than 0.90 indicating, respectively, moderate, good and excellent reliability.

# **Table S4.** Residuals corrected for age of relative regional brain atrophy differences among groups classified according to the density of neocortical neuritic plaques (CERAD score).

| **Corrected age** | **PART** | **AD** | | |  |
| --- | --- | --- | --- | --- | --- |
|  | **CERAD 0** (None)  (n = 29) | **CERAD 1** (Sparse)  (n = 17) | **CERAD 2** (Moderate)  (n = 54) | **CERAD 3** (Severe)  (n = 67) | *p* |
| AC | -0.155 (±0.772) | 0.355 (±1.130) | -0.193 (±0.717) | 0.128 (±0.918) | 0.124 |
| OF | 0.185 (±0.725) | -0.208 (±1.052) | 0.087 (±0.818) | -0.092 (±0.913) | 0.268 |
| AT | 0.085 (±0.584) | -0.347 (±0.700) | -0.051 (±1.038) | 0.0909 (±0.855) | 0.320 |
| FI | 0.197 (±0.896) | -0.438 (±0.808) | -0.199 (±0.821) | 0.187 (±0.857) | **0.022** |
| MTA | -0.191 (±0.754) | -0.346 (±0.898) | -0.089 (±0.931) | 0.236 (±0.815) | **0.044** |
| Post | 0.043 (0.823) | -0.103 (±0.899) | -0.227 (±0.784) | 0.188 (±0.922) | 0.099 |

The table shows the assessment of potential differences between the residuals corrected for age of relative regional brain atrophy among groups classified according to the CERAD score, by using Kruskal-Wallis tests. The residuals are represented by mean (±standard deviation) for each group, and significant differences with the *p*-value in **bold**. p < 0.05 considered as statistically significant.
